# Effects of between-person differences and within-person changes in symptoms of anxiety and depression on older age cognitive performance

**DOI:** 10.1017/S0033291717002896

**Published:** 2017-10-17

**Authors:** E. J. Laukka, D. Dykiert, M. Allerhand, J. M. Starr, I. J. Deary

**Affiliations:** 1 Department of Neurobiology, Care Sciences, and Society (NVS), Aging Research Center, Karolinska Institutet and Stockholm University, Stockholm, Sweden; 2 Centre for Cognitive Ageing and Cognitive Epidemiology, University of Edinburgh, Edinburgh, UK; 3 Department of Psychology, University of Edinburgh, Edinburgh, UK; 4 Geriatric Medicine Unit, University of Edinburgh, Edinburgh, UK

**Keywords:** Anxiety, aging, cognitive function, depression, population-based study, within-person change

## Abstract

**Background:**

Anxiety and depression are both important correlates of cognitive function. However, longitudinal studies investigating how they covary with cognition within the same individual are scarce. We aimed to simultaneously estimate associations of between-person differences and within-person variability in anxiety and depression with cognitive performance in a sample of non-demented older people.

**Methods:**

Participants in the Lothian Birth Cohort 1921 study, a population-based narrow-age sample (mean age at wave 1 = 79 years, *n* = 535), were examined on five occasions across 13 years. Anxiety and depression were measured with the Hospital Anxiety and Depression Scale (HADS) and cognitive performance was assessed with tests of reasoning, logical memory, and letter fluency. Data were analyzed using two-level linear mixed-effects models with within-person centering.

**Results:**

Divergent patterns were observed for anxiety and depression. For anxiety, between-person differences were more influential; people who scored higher on HADS anxiety relative to other same-aged individuals demonstrated poorer cognitive performance on average. For depression, on the other hand, time-varying within-person differences were more important; scoring higher than usual on HADS depression was associated with poorer cognitive performance relative to the average level for that participant. Adjusting for gender, childhood mental ability, emotional stability, and disease burden attenuated these associations.

**Conclusions:**

The results from this study highlight the importance of addressing both between- and within-person effects of negative mood and suggest that anxiety and depression affect cognitive function in different ways. The current findings have implications for assessment and treatment of older age cognitive deficits.

## Introduction

General cognitive ability is a stable trait with high intraindividual correlations across the life span. Although some aspects of cognitive function generally decline with age, there are large individual differences in older age cognitive performance. This variability can be due to differences that were present during most of life (Deary *et al.* 2013; Deary & Brett, 2015), or to differences in the rate of age-related decline (de Frias *et al.* 2007; Small *et al.* 2011). Furthermore, cognitive performance may vary within the same individual, from one occasion to another, for reasons other than age. These fluctuations may be partly due to random factors; however, previous research has shown that they can be explained and thus systematically investigated (Nesselroade & Salthouse, 2004; Sliwinski *et al.* 2006). Several factors have been associated with this intraindividual variability, such as motivation, well-being, stress, and positive and negative affect (Sliwinski *et al.* 2006; Brose *et al*. 2010, 2012, 2014; Allerhand *et al.* 2014). To achieve fuller understanding of older adults’ cognitive performance, both between-person differences and within-person variability need to be taken into account.

Depression, even in its mildest forms, has been associated with lower levels of cognitive function. People with clinical depression (Pantzar *et al.* 2014), depressive symptoms (Dotson *et al.* 2008), or experimentally induced low mood (Seibert & Ellis, 1991) on average perform worse on cognitive tests compared to individuals free of depression. A range of domains, including memory (Dotson *et al.* 2008), perceptual speed (Bielak *et al.* 2011), and verbal fluency (Freiheit *et al.* 2012) are affected, and having more depressive symptoms have been associated with both lower performance and faster cognitive decline in older adults. Studies on the effects of anxiety on older age cognitive performance have produced more mixed results. Cross-sectionally, anxiety has been related to poorer performance, whereas few significant associations with the rate of cognitive decline have been observed (Bierman *et al.* 2008; Beaudreau & O'Hara, 2009; Bunce *et al.* 2012; de Bruijn *et al.* 2014).

Symptoms of anxiety and depression show large degrees of stability across the lifespan, mainly due to genetic influences (Johnson *et al.* 2002; Nivard *et al.* 2015), but may vary across adulthood due to, for example, environmental factors, positive and negative life events, and protective and risk factors accumulating over time (Rosenström *et al.* 2013). Longitudinal studies offer the possibility of assessing not only between-person differences but also whether two variables are coupled together and covary within the same individual. However, most previous studies have limited their investigations to between-person relationships, and findings from studies investigating correlated change in depressive symptoms and cognition have not been consistent (van den Kommer *et al.* 2013; Brailean *et al.* 2017). Separating between-person and within-person effects provides important complementary information as they may sometimes give different or even opposite patterns of results (Curran & Bauer, 2011; Thorvaldsson *et al.* 2012).

Here, we aimed to explore the influences of time-invariant between-person differences and time-varying within-person changes in negative affect (anxiety and depression) on cognitive performance in a sample of non-demented older adults. At the between-person level, we estimate the effects of having a higher level of anxiety or depression compared to other individuals of the same age. At the within-person level, we address the effects of an individual deviating from their average level of depression or anxiety. Further, we explore the moderating role of known correlates to cognitive performance, anxiety, and depression on these associations.

## Methods

### Participants

Data were collected from the Lothian Birth Cohort 1921 (LBC1921) sample. Recruitment and data collection in this study have been described in detail elsewhere (Deary *et al.* 2004, 2012). In brief, LBC1921 follows up older people resident in Edinburgh or the Lothians, Scotland, who were born in 1921. At age 11, most participants were tested with a general intelligence test as part of the Scottish Mental Survey of 1932 (Scottish Council for Research in education, 1933). Participants were recruited for a follow-up study in 1999–2001 at a mean age of about 79 years. The 550 participants tested at wave 1 were later invited back for follow-up assessments at a mean age of 83 (wave 2, *n* = 321), 87 (wave 3, *n* = 235), 90 (wave 4, *n* = 129), and 92 (wave 5, *n* = 59) years. Reasons for attrition were death (*n* = 208), severe illness (*n* = 129), refusal (*n* = 75), loss of contact (*n* = 16), or other (*n* = 63). Each assessment involved an interview, cognitive testing, a physical examination, and self-report questionnaires. Ethics permissions were obtained from the Lothian (waves 1–3) and the Scotland A (waves 4 and 5) Research Ethics Committees. Informed consent was collected from all participants and the ethical guidelines from the Declaration of Helsinki were followed.

For the present analyses, we excluded participants who had a self-reported history of dementia or a Mini-Mental State Examination (MMSE: Folstein *et al.* 1975) score < 24 (*n* = 13), or missing data on the Hospital Anxiety and Depression Scale (HADS: Zigmond & Snaith, 1983, *n* = 2) at wave 1, resulting in a sample of 535 persons. For the follow-up assessments, we excluded participants with missing data on HADS; participants with a record of dementia history or low MMSE score were excluded for that and all subsequent waves. After these exclusions, follow-up data were available for 302 participants at wave 2, 187 participants at wave 3, 111 participants at wave 4, and 50 participants at wave 5; 233 participants contributed with data only at wave 1, whereas 115 participants had data for two waves, 76 for three, 61 for four, and 50 for all five waves. Mean follow-up time between waves was 4.32, 3.25, 3.46, and 2.01 years, respectively.

### Measures

#### Cognitive abilities

Childhood mental ability was assessed at age 11 with a modified version of the Moray House Test No. 12. This is a test of general intelligence that was validated against the Terman–Merill revision of the Binet scales (Scottish Council for Research in Education, 1933) and scaled to provide IQ-type scores.

At each wave of the LBC1921 study, participants completed a battery of cognitive tests. Here, we report on the tests that were administered at wave 1 and all four subsequent waves.

*Raven's Standard Progressive Matrices* (Raven *et al.* 1977) was used to assess non-verbal reasoning ability. For this 60-item test, participants were asked to choose the correct item from a set of alternatives, to complete an incomplete pattern. The score used was number of correctly completed patterns within the 20 min time limit.

The *Logical Memory* subtest of the Wechsler Memory Scale (Wechsler, 1987) measures verbal declarative memory. Participants had two short stories read out loud to them. Immediately after each reading, and again after a minimum of 30 min delay, they were asked to tell the interviewer as much as they could remember from each story. The score used was total number of correctly recalled memory elements from immediate and delayed recall for the two stories combined.

Three *Letter fluency* (Lezak, 2004) tasks were included as measures of verbal fluency ability. Here, participants were asked to generate as many words as possible beginning with the letters C, F, and L, with a time limit of 1 min for each letter. The score used was total number of words generated across the three tasks.

#### Symptoms of anxiety and depression

HADS was developed by Zigmond & Snaith (1983) to identify cases of anxiety and depression among patients in non-psychiatric hospital clinics. This self-assessment questionnaire includes 14 items, seven for anxiety and seven for depression, rated on a scale from 0 to 3. Participants are instructed to underline the alternative that best reflects how they have been feeling in the past week. Anxiety and depression are generally considered separately and maximum score on each subscale is 21. A score of 0–7 is considered normal; 8–10 is considered to be an indication of mild anxiety/depression; whereas scores ⩾11 indicate anxiety/depression. Both HADS subscales show good internal consistency and concurrent validity (Bjelland *et al.* 2002). In LBC1921, HADS was administered at waves 1, 3, 4, and 5.

#### Emotional stability

Personality was measured at wave 1 using the International Personality Item Pool Big-Five 50-item inventory (Goldberg, 1992). This scale has 10 items for each of the Big-Five personality factors and has previously been validated in LBC1921 (Gow *et al.* 2005). Emotional stability represents the same trait as neuroticism but is named and scored from the opposite end of the continuum, with higher scores representing more emotional stability.

#### Disease burden

A medical history, including diagnoses of cancer, cerebrovascular, and cardiovascular diseases, was taken as a part of a standardized interview at wave 1. Disease burden was summarized as number of diseases.

### Statistical analyses

We used two-level linear mixed-effects models with within-person centering (Hoffman & Stawski, 2009; Allerhand *et al.* 2014), which allow the separation of time-varying within-person effects from time-invariant between-person effects of anxiety and depression upon cognitive function.

Initially, six mixed-effects models with random intercept were fitted: models of reasoning, memory, and fluency each predicted by either anxiety or depression scores. These basic models were unadjusted except for age (centered on 79 years, being the mean age at wave 1). In each model, the time-varying predictor (anxiety or depression) was replaced by two independent variables derived from it: a within-person centered (WP) variable and a person mean (PM) variable. The WP variable was entered as a time-varying (level-1) variable. The PM variable was centered on its mean, and entered as a time-invariant (level-2) variable in the equation for the random intercept (the PM main effect). The models included their cross-level interaction. The models were fitted by R function lmer (package lme4). Residuals were assessed graphically for normality and judged to be acceptable. Following this, four covariates were added to each model: gender, age 11 IQ, emotional stability, and number of diseases. These were all centered on their means, except gender which was coded such that 0 reflects male and 1 reflects female gender. The covariates were first added individually to assess the effect of each by itself. The final, fully adjusted models included the main effects of the within- and between-person centered anxiety or depression variables and their interactions with each other and with age, and the main effects of the four covariates and their interactions with the within- and between-person centered variables.

## Results

Characteristics of the study sample according to wave are shown in Table 1. Levels of anxiety and depression in this sample were generally low. Of the 121 individuals (23%) who were above the frequently used cut off for anxiety (⩾8) at wave 1, 92 (76%) had mild anxiety and 29 (24%) moderate-to-severe anxiety. Of the 34 persons (6%) above the cut off for depression (⩾8), 29 (85%) had mild and only five (15%) moderate-to-severe depression. At waves 1 and 3, participants tended to score higher on anxiety than on depression (p < 0.05), whereas these scores were not significantly different at waves 4–5. This could be due to higher dropout rates among persons scoring high on anxiety at wave 1 or increasing depression scores with increasing age. For characteristics of those who stayed in the study for the entire follow-up (i.e. completers; *n* = 50), see online Supplementary Table S1. Completers had significantly fewer diseases and better cognitive performance at wave 1 compared to non-completers (p < 0.05).

**Table 1.** Sample characteristics according to wave

	Wave 1 (*n* = 535)		Wave 2 (*n* = 302)		Wave 3 (*n* = 187)		Wave 4 (*n* = 111)		Wave 5 (*n* = 50)	
	Mean	s.d.	Mean	s.d.	Mean	s.d.	Mean	s.d.	Mean	s.d.
Age	79.12	0.58	83.41	0.55	86.68	0.42	90.18	0.15	92.16	0.36
Gender, % female	57.76									
Age 11 IQ, *n* = 479	100.33	14.75								
Emotional stability, *n* = 442	24.31	8.12								
No. of diseases, *n* = 516	1.58	1.55								
HADSa (anxiety)	5.19	3.30			4.43	3.16	3.77	2.92	5.06	3.09
HADSd (depression)	3.50	2.30			3.86	2.42	3.86	2.28	4.72	2.45
HADSa: deviation from within-person mean	0.03	0.27			−0.02	0.49	−0.13	0.39	0.06	0.44
HADSd: deviation from within-person mean	−0.09	0.37			0.05	0.57	0.14	0.60	0.41	0.63
Raven's^a^	31.50	8.55	30.43	8.81	28.55	8.82	26.84	8.23	26.00	8.18
Logical memory^b^	31.96	12.65	33.83	14.10	34.44	13.93	35.27	16.19	37.24	18.68
Letter fluency^c^	40.24	12.27	40.29	12.78	40.86	11.93	40.16	13.34	40.82	12.98

s.d., standard deviation; HADS, Hospital Anxiety and Depression Scale.

a Number of participants with data on Raven's were 528 at wave 1, 298 at wave 2, 183 at wave 3, 105 at wave 4, and 46 at wave 5.

b Number of participants with data on Logical memory were 535 at wave 1, 301 at wave 2, 187 at wave 3, 111 at wave 4, and 50 at wave 5.

c Number of participants with data on Letter fluency were 532 at wave 1, 301 at wave 2, 187 at wave 3, 111 at wave 4, and 50 at wave 5.

**Fig. 1.**
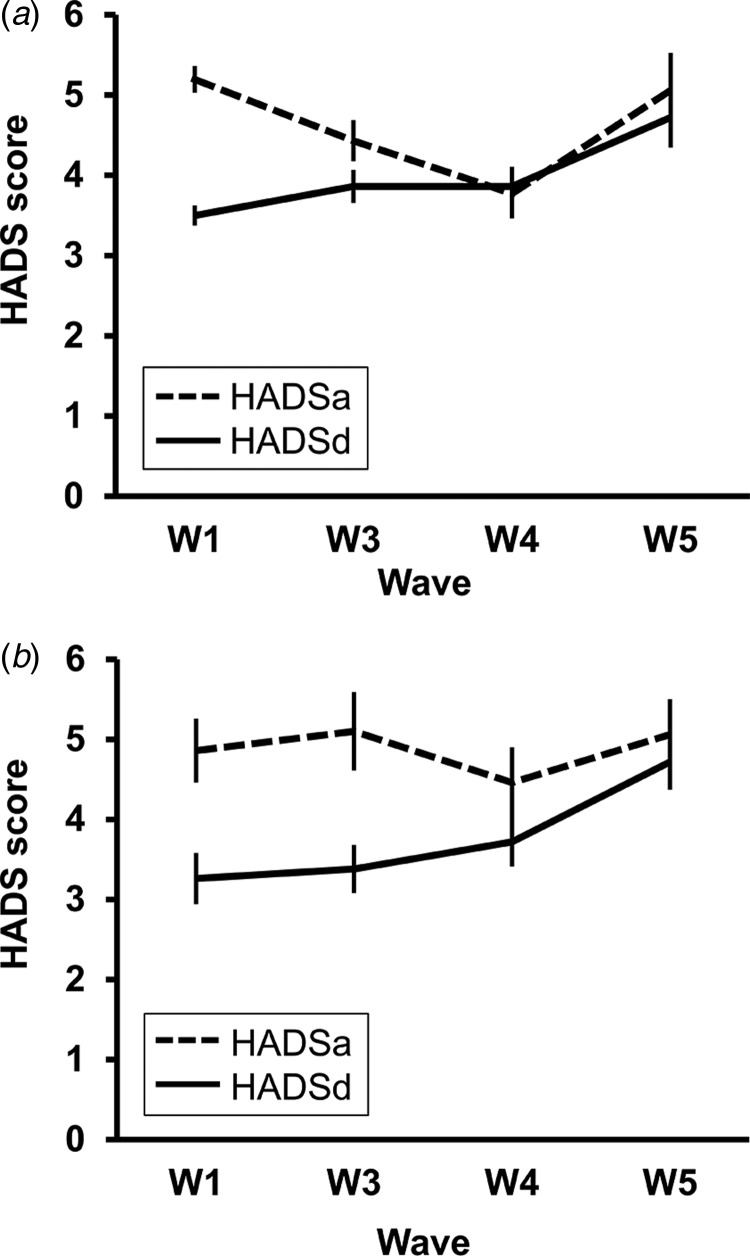
Levels of anxiety and depression across waves for the total sample (*a*; *n* = 535) and for individuals who stayed in the study for the entire follow-up period (*b*; *n* = 50).

Figure 1 presents an overview of HADS scores across waves for the total sample and for completers. HADS anxiety scores assessed at wave 1 were highly correlated with anxiety scores at subsequent waves (total sample: wave 3: 0.57, wave 4: 0.69, wave 5: 0.70; completers: wave 3: 0.61, wave 4: 0.68, wave 5: 0.70, p < 0.001). HADS depression scores at wave 1 showed somewhat weaker correlations with subsequent depression scores (total sample: wave 3: 0.43, wave 4: 0.39, wave 5: 0.53; completers: wave 3: 0.67, wave 4: 0.50, wave 5: 0.53, p < 0.001). Again, this might be partly due to increasing depression scores with each wave, but it could also indicate greater variability for the depression scores. Formally comparing the correlations, however, they only differed significantly for wave 4 (*p* < 0.01, total sample).

We calculated the within-person mean and the within-person standard deviation (s.d.) across all four waves for HADS anxiety (mean = 5.10, s.d. = 1.61) and HADS depression (mean = 3.70, s.d. = 1.46). Correlations among the covariates and within-person mean and s.d. for anxiety and depression are shown in Table 2. Having a higher score on HADS anxiety was related to lower age 11 IQ, less emotional stability, and a larger number of diseases; women tended to score higher on HADS anxiety. HADS depression was negatively associated with emotional stability. There was a positive association between anxiety and depression, so that persons scoring higher on HADS anxiety tended to score higher on HADS depression, and *vice versa*. Higher variability in anxiety was related to less emotional stability; similarly, higher variability in depression was related to less emotional stability and having more diseases. Furthermore, higher levels (mean) of anxiety or depression were related to more variability (s.d.).

**Table 2.** Correlations among covariates, mean anxiety and depression, and variability in anxiety and depression across waves

	Gender^a^	Age 11 IQ	Emotional stability	No. of diseases	HADS anxiety (mean)	HADS depression (mean)	HADS anxiety (s.d.)
Age 11 IQ	0.03						
Emotional stability	−0.05	0.11*					
No. of diseases	−0.04	−0.04	−0.08				
HADS anxiety (mean)	0.18***	−0.10*	−0.53***	0.10*			
HADS depression (mean)	−0.02	0.00	−0.31***	0.04	0.37***		
HADS anxiety (s.d.)	0.01	−0.14	−0.28***	0.12	0.26***	0.18*	
HADS depression (s.d.)	−0.01	−0.03	−0.16*	0.21**	0.20**	0.43***	0.15*

s.d., standard deviation; HADS, Hospital Anxiety and Depression Scale.

a Associations with gender were analyzed using point-biserial correlation.

*p < 0.05, **p < 0.01, ***p < 0.001.

The results from the main analyses are shown in Table 3. Here we report the associations between the outcome (Raven's, Logical memory, or Letter fluency) and the focal predictor (anxiety or depression), represented by the WP and PM variables, their interactions with each other, and their interactions with age. The WP effect reflects the difference from a participant's usual cognitive score per within-person s.d. increase in anxiety/depression relative to the average level for that participant. In other words, it tells us how much within-person changes in mood affect the person's cognitive performance. The PM effect, on the other hand, is a between-person effect. It reflects the difference in cognitive score between people whose mean level of anxiety/depression differs by 1 s.d.

**Table 3.** Associations between HADS anxiety and depression scores and performance on cognitive tests (fixed effects)

	Raven's		Logical memory		Letter fluency	
	Basic model	Fully adjusted model	Basic model	Fully adjusted model	Basic model	Fully adjusted model
Anxiety						
Age	−0.0772 (0.0050)***	−0.0837 (0.0051)***	0.0049 (0.0059)	0.0005 (0.0065)	−0.0168 (0.0047)***	−0.0232 (0.0052)***
WP	0.1811 (0.0977)	0.0844 (0.1279)	−0.0122 (0.1166)	−0.0402 (0.1598)	0.1007 (0.0926)	−0.0026 (0.1283)
PM	−0.2059 (0.0450)***	−0.0797 (0.0848)	−0.0931 (0.0476)	−0.0029 (0.1007)	−0.0974 (0.0453)*	−0.1637 (0.0919)
WP x age	−0.0180 (0.0139)	−0.0044 (0.0150)	0.0140 (0.0166)	0.0141 (0.0187)	−0.0046 (0.0132)	−0.0050 (0.0151)
PM x age	0.0106 (0.0059)	0.0080 (0.0064)	0.0125 (0.0070)	0.0169 (0.0080)*	0.0073 (0.0056)	0.0014 (0.0065)
WP x PM	0.0208 (0.0627)	−0.0308 (0.0836)	−0.0426 (0.0743)	−0.1657 (0.1021)	−0.0664 (0.0575)	−0.1002 (0.0811)
Depression						
Age	−0.0757 (0.0051)***	−0.0804 (0.0052)***	0.0055 (0.0060)	−0.0013 (0.0066)	−0.0129 (0.0048)**	−0.0160 (0.0053)**
WP	−0.1410 (0.0795)	−0.0382 (0.0910)	−0.2116 (0.0944)*	−0.2136 (0.1143)	−0.2195 (0.0736)**	−0.2261 (0.0913)*
PM	−0.0754 (0.0456)	0.0960 (0.0773)	0.0148 (0.0477)	0.1042 (0.0929)	−0.0748 (0.0454)	−0.1766 (0.0837)*
WP x age	0.0096 (0.0101)	0.0045 (0.0100)	0.0222 (0.0115)	0.0181 (0.0126)	0.0118 (0.0091)	0.0061 (0.0102)
PM x age	−0.0009 (0.0065)	−0.0040 (0.0067)	0.0040 (0.0075)	0.0053 (0.0081)	−0.0067 (0.0060)	−0.0070 (0.0066)
WP x PM	−0.0180 (0.0497)	−0.0292 (0.0519)	−0.0644 (0.0579)	−0.0996 (0.0637)	0.0248 (0.0442)	0.0068 (0.0504)

HADS, Hospital Anxiety and Depression Scale.

Estimates are in standard deviation (s.d.) units, with standard errors in brackets. WP is the effect of the average person deviating 1 s.d. unit from their own within-person mean score on the focal predictor (anxiety or depression). PM is the effect of the average individual scoring 1 s.d. unit higher on the person-mean variable of the focal predictor (anxiety or depression). Estimates labeled a x b are interactions. The fully adjusted model controls for gender, age11 IQ, emotional stability, and number of diseases. All WP x covariate and PM x covariate interactions were included in the models, but are not included in the table. Significance was based on standard errors.

*p < 0.05, **p < 0.01, ***p < 0.001.

Different patterns were observed for anxiety and depression. Specifically, for anxiety, PM effects appeared to be more important, whereas for depression, it was mainly the WP effect that was linked to cognitive outcomes. The cross-level interaction between WP and PM was non-significant in all models except one, suggesting that the effect of within-person changes in anxiety/depression was independent of the person's level of anxiety/depression, and *vice versa*.

Let us consider PM (i.e. between-person) effects first. For anxiety, people who scored higher on HADS on average had poorer cognitive performance. After accounting for age and the WP effect, scoring 1 s.d. higher on HADS anxiety compared to the population mean was associated with 0.21 s.d. lower performance on Raven's, 0.09 s.d. lower performance on Logical memory and 0.10 s.d. lower performance on Letter fluency. The association was statistically significant for Raven's (*p* < 0.001) and Letter fluency (*p* < 0.05), whereas it was marginally significant for Logical memory (*p* = 0.05, basic model). Controlling for the covariates weakened these associations, and in the fully adjusted models, they were no longer significant. For a full report on all models, including the effect of each covariate, see online Supplementary Tables S2–S4. PM effects of depression were not significant for any of the cognitive outcomes in basic models.

Turning now to the WP (i.e. within-person) effects, having a higher than usual depression score on a testing occasion, was associated with poorer cognitive performance on that occasion. After adjusting for age and PM effects, the WP effect was significant for Logical memory (*p* < 0.05) and Letter fluency (*p* < 0.01), and at a non-significant trend level for Raven's (*p* = 0.07, basic model). For Logical memory, the association was no longer significant after controlling for covariates, whereas the association remained significant in the fully adjusted model for Letter fluency. For this task, there was also a significant effect of between-person difference in depression symptoms, suggesting that PM and WP effects of depression both gave independent contributions to performance on Letter fluency. The between-person effect only appeared after adding gender as covariate. In the fully adjusted model, a person scoring 1 s.d. higher than the group mean on HADS depression was performing on average 0.18 s.d. poorer on Letter fluency. Furthermore, scoring 1 s.d. higher on HADS depression relative to that individual's usual level was associated with 0.23 s.d. lower performance on Letter fluency relative to that person's mean level. No WP anxiety effects were significant for any of the cognitive outcomes.

For every year increase in age, participants showed significant decline on Raven's and Letter fluency, but not on Logical memory. There were only a few cases in which the interactions between age and the WP and PM variables were significant, suggesting that these effects were largely age-invariant. In all cases where an interaction was observed, the interaction suggested that the negative WP or PM effect weakened with age.

Effects of single covariates are shown in online Supplementary Tables S2–S4. Females showed poorer performance on Raven's, age 11 IQ was positively associated with all cognitive outcomes, emotional stability was positively associated with all cognitive outcomes in the models for depression, and number of diseases was negatively associated with performance on Logical memory and Letter fluency. There were some significant interactions between the WP and PM variables and the covariates. Scoring higher on emotional stability was associated with a larger negative WP effect in the depression model for Logical memory, although this interaction effect was not significant in the fully adjusted model. Having more diseases at wave 1 was associated with a smaller negative PM effect in the depression model for Letter fluency. In contrast, higher disease burden was associated with a smaller positive WP effect in the anxiety model and a larger negative WP effect in the depression model for Raven's.

## Discussion

The results of this study show that both anxiety and depression are negatively associated with cognitive performance, which is in agreement with previous research. However, a novel finding is that separating the between-person and within-person influences on cognitive function resulted in different patterns of results for anxiety and depression; whereas between-person differences showed to be more important for anxiety, within-person changes appeared more influential for depression.

The observed pattern for anxiety confirms the results of previous studies on the cross-sectional influence of anxiety on cognition (Beaudreau & O'Hara, 2009; Bunce *et al.* 2012). In comparison, within-person variability in anxiety seems to have a smaller effect. This suggests that, after controlling for between-person effects, within-person variability in anxiety is not an important factor for older age cognitive performance. It should be noted, however, that the variability in anxiety was relatively modest; for people who stayed in the study, anxiety levels remained rather stable across waves (*r* = 0.61–0.70). As increases were relatively small and likely to be below the clinical threshold in most cases, they might not have been large enough to affect cognitive performance. Furthermore, anxiety might even be beneficial in a testing situation, up to a certain point. Results from a previous study on older adults are in support of such a threshold effect; mild anxiety symptoms were associated with better cognitive performance and it was only with increasing levels that a negative effect appeared (Bierman *et al.* 2005).

Anxiety levels have been found to be more stable across time within elderly people compared to levels of depression (Wetherell *et al.* 2001). Possibly, this reflects that anxiety is closely related to underlying personality traits. Associations between anxiety and neuroticism are typically high (Watson & Clark, 1984), as confirmed by the present study. The genetic influence on the variance in anxiety increases from age 60 at the expense of environmental influences (Lee *et al.* 2016), thus further supporting the view of older age anxiety as a more trait-like characteristic. In addition to being more stable, older individuals may be less affected by changes in anxiety compared to young adults. In a study comparing two age groups, older adults both reported experiencing fewer stressors and that the stressors had less impact on their daily routines (Brose *et al.* 2013).

The largest effect of anxiety on cognition was observed for Raven's, a test of reasoning ability. This is consistent with Eysenck's processing efficiency theory (Eysenck & Calvo, 1992), stating that anxiety interferes with cognitive tasks by reducing available resources for working memory. Therefore, tasks relying on the central executive of working memory, for example, problem solving and reasoning tasks, are hypothesized to be mostly affected by anxiety. Older adults might be particularly vulnerable to such effects as their working memory capacity is already limited.

More anxiety symptoms were observed in individuals with lower age 11 IQ, females, and persons with lower emotional stability (Watson & Clark, 1984; Johnson *et al.* 2011; Lee *et al.* 2016). Covarying all of these resulted in non-significant effects of anxiety for Raven's. Childhood mental ability showed the strongest association to cognition (Deary *et al.* 2013) and was also related to anxiety; controlling for this variable resulted in non-significant associations between anxiety and Letter fluency. For Logical memory, effects of anxiety were only borderline significant (*p* = 0.05) but followed the same trend as for the other cognitive tasks, with between-person effects being most important.

In contrast to the results for anxiety, the results for depression suggest that within-person variability is more important for cognition in the elderly population compared to differences in mean level. That depression is negatively related to cognitive performance is in agreement with the bulk of research in this area (Dotson *et al.* 2008; Köhler *et al.* 2010). With regard to longitudinal associations, a large population-based study reported that the course of cognitive functioning was not significantly associated with the course of depressive symptoms (van den Kommer *et al.* 2013), whereas a recent study observed that steeper decline in processing speed correlated with a steeper increase in depressive symptoms (Brailean *et al.* 2017). In a sample of older adults with major depressive disorder, having more depressive symptoms than usual was associated with worse than average global cognitive function (Dzierzewski *et al.* 2015). Here, we provide evidence that depressive symptoms covary with cognitive performance, especially letter fluency, in the general elderly population.

When interpreting these findings, one should bear in mind that very few participants had clinical depression. To have a current diagnosis of depression, or having experienced several reoccurring depressive episodes, is still likely to have an impact on cognitive performance at the between-person level. We observed associations at the between- as well as the within-person level between for Letter fluency. This is consistent with previous findings that tasks depending on frontal lobe functioning is affected already in mild depression, and may also persist in remission (Köhler *et al.* 2010; Pantzar *et al.* 2014).

An important finding of this study is that even rather modest intraindividual change in depression exerts significant influence on cognitive performance in the general population. Thus, our results extend the findings from a previous study targeting a population of depressed individuals (Dzierzewski *et al.* 2015). The observed effects may reflect changes due to environmental factors, or feeling more happy or sad on a particular day. However, they may also reflect the common trend of increasing levels of depressive symptoms after age 60 (Johnson *et al.* 2002; Chui *et al.* 2015); mean levels of depression increased with increasing age. The combined pattern from this study suggests that variability in depressive symptoms is more important for cognitive functioning than within-person changes in anxiety. This corroborates previous findings that depressive symptoms show a linear association with cognition, where more symptoms are associated with worse cognition, whereas anxiety levels need to exceed a certain threshold before exerting a negative influence on cognitive performance (Bierman *et al.* 2005).

The strongest effects for depression were observed for Letter fluency. This is in agreement with that the most consistent associations between depression and cognition have been observed for tasks dependent on speed and executive functioning (Köhler *et al.* 2010; Pantzar *et al.* 2014); Letter fluency requires both fast processing and active generation of responses. Logical memory also covaried with number of depressive symptoms. However, controlling for age 11 IQ, emotional stability, or number of diseases resulted in non-significant associations. Results were somewhat weaker for Raven's (*p* = 0.07) and largely disappeared after controlling for gender. Cross-level interactions between WP and PM were not significant, meaning that within-person associations with cognition were not different for individuals with high or low levels of depression.

There are several potential explanations for the association between depression and cognitive performance. Feeling more depressed than usual may place higher demands on available resources (Seibert & Ellis, 1991) and negative affect has been shown to covary inversely with motivation (Brose *et al.* 2012). These factors may contribute to the intraindividual coupling of depression and cognition. In addition, clinical depression may have more long-term effects on cognition by causing structural and functional brain changes (Duman *et al.* 1997). However, the direction of potential causal relationships is not clear and it might also be that a third factor, for example, disease burden or impending dementia, accounts for the association. Depressive symptoms may be both risk factors or early markers of dementia (Byers & Yaffe, 2011), and it is possible that subclinical neurodegenerative or vascular changes underlie both the depressive symptoms and cognitive declines observed in this study.

## Strengths and limitations

A strength of the present study is that we were able to assess both between- and within-person effects of both anxiety and depression within the same individuals. Additional strengths are that we could investigate these effects in a large population-based sample with information on performance in several cognitive domains as well as important confounding variables. The fact that all individuals were of nearly the same age at wave 1 and that we controlled for longitudinal age effects in the analyses make it possible to disregard the effects of age in this study, which would have been a major possible confounder.

The study also has limitations; one is that we do not have information on levels of depression and anxiety between assessments. Given the relatively long time intervals between testing occasions, our findings are likely to inform on general trends spanning longer time periods rather than the effects of day-to-day fluctuation in mood. It is also probable that there was some selective attrition throughout the study, restricting the range for anxiety and depression (see Results section, first paragraph). Our findings are correlational and thus do not inform on the direction of the association or any potential causal relationships.

## Implications

Symptoms of anxiety and depression are common in old age. This study confirms that they are both important correlates of cognition but also shows that they affect performance in different ways. For anxiety, mean levels were more important, strengthening the view of anxiety as a more trait-like factor in relation to cognitive performance. For depression, deviating from one's mean level was more important, illustrating that even rather small changes in depression may affect cognitive performance. The results have implications for the interpretation of cognitive test scores, as using scores based on only one occasion may give a somewhat misleading picture of that individual's actual cognitive abilities. The fluctuating nature of depression and its impact on cognitive performance may explain some of the inconsistencies in previous research concerning depressive symptoms and cognitive decline (Han *et al.* 2006; Dzierzewski *et al.* 2015). From a treatment perspective, the results from this study also show that when levels of depression go down, cognitive performance improves. This suggests that reducing levels of depressive symptoms, even outside the clinical range, may lead to improved cognitive performance.

## Supplementary material

For supplementary material accompanying this paper visit http://dx.doi.org/10.1017/S0033291717002896. click here to view supplementary material
