# Supplementary material for: Effects of between-person differences and within-person changes in symptoms of anxiety and depression on older age cognitive performance
Source: Psychol Med. 2017 Oct 17;48(8):1350–8. doi: 10.1017/S0033291717002896 (PMC6088541; doi:10.1017/S0033291717002896)
Supplement: Supplementary file 1 [file S0033291717002896sup.zip › S0033291717002896sup002.docx]

**Effects of between-person differences and within-person fluctuations in symptoms of anxiety and depression on older-age cognitive performance**

**Table 2S.** Associations between HADS anxiety and depression scores and performance on Raven’s (fixed effects)

|  | Basic model | Adjusted for  gender | Adjusted for  age 11 IQ | Adjusted for emotional stability | Adjusted for  no. of diseases | Fully adjusted model |
| --- | --- | --- | --- | --- | --- | --- |
| **Anxiety** |  |  |  |  |  |  |
| Age | -0.0772 (0.0050)*** | -0.0773 (0.0050)*** | -0.0833 (0.0051)*** | -0.0789 (0.0051)*** | -0.0782 (0.0051)*** | -0.0837 (0.0051)*** |
| WP | 0.1811 (0.0977) | 0.1876 (0.1186) | 0.1190 (0.1002) | 0.1564 (0.1006) | 0.1136 (0.1004) | 0.0844 (0.1279) |
| PM | **-0.2059 (0.0450)***** | **-0.1530 (0.0728)*** | **-0.1637 (0.0439)***** | **-0.1974 (0.0587)***** | **-0.2065 (0.0461)***** | -0.0797 (0.0848) |
| WP*age | -0.0180 (0.0139) | -0.0178 (0.0141) | -0.0110 (0.0145) | -0.0144 (0.0141) | -0.0117 (0.0144) | -0.0044 (0.0150) |
| PM*age | 0.0106 (0.0059) | 0.0104 (0.0059) | 0.0056 (0.0063) | 0.0112 (0.0061) | 0.0126 (0.0059)* | 0.0080 (0.0064) |
| WP*PM | 0.0208 (0.0627) | 0.0224 (0.0633) | -0.0352 (0.0690) | 0.0366 (0.0729) | 0.0251 (0.0629) | -0.0308 (0.0836) |
| WP*no. of diseases |  |  |  |  |  | -0.0847 (0.0342)* |
| Gender (female) |  | -0.2060 (0.0863)* |  |  |  | -0.1755 (0.0878)* |
| Age 11 IQ |  |  | 0.0281 (0.0027)*** |  |  | 0.0282 (0.0030)*** |
| Emotional stability |  |  |  | 0.0094 (0.0066) |  | 0.0120 (0.0064) |
| No. of diseases |  |  |  |  | -0.0058 (0.0277) | 0.0007 (0.0283) |
| **Depression** |  |  |  |  |  |  |
| Age | -0.0757 (0.0051)*** | -0.0756 (0.0051)*** | -0.0806 (0.0052)*** | -0.0777 (0.0051)*** | -0.0762 (0.0052)*** | -0.0804 (0.0052)*** |
| WP | -0.1410 (0.0795) | -0.0893 (0.0926) | -0.1184 (0.0781) | -0.1188 (0.0788) | -0.1361 (0.0793) | -0.0382 (0.0910) |
| PM | -0.0754 (0.0456) | -0.0449 (0.0700) | -0.0744 (0.0432) | 0.0008 (0.0534) | -0.0664 (0.0463) | 0.0960 (0.0773) |
| WP*age | 0.0096 (0.0101) | 0.0086 (0.0101) | 0.0089 (0.0101) | 0.0066 (0.0101) | 0.0083 (0.0101) | 0.0045 (0.0100) |
| PM*age | -0.0009 (0.0065) | -0.0022 (0.0066) | -0.0010 (0.0066) | -0.0008 (0.0066) | 0.0004 (0.0066) | -0.0040 (0.0067) |
| WP*PM | -0.0180 (0.0497) | -0.0168 (0.0497) | -0.0432 (0.0495) | -0.0301 (0.0517) | -0.0072 (0.0494) | -0.0292 (0.0519) |
| WP*no. of diseases |  |  |  |  | -0.0788 (0.0248)* | -0.0518 (0.0251)* |
| Gender (female) |  | -0.2598 (0.0856)** |  |  |  | -0.1710 (0.0858)* |
| Age 11 IQ |  |  | 0.0296 (0.0028)*** |  |  | 0.0297 (0.0030)*** |
| Emotional stability |  |  |  | 0.0173 (0.0060)** |  | 0.0154 (0.0058)** |
| No. of diseases |  |  |  |  | -0.0080 (0.0282) | 0.0074 (0.0282) |

HADS, Hospital Anxiety and Depression Scale.

Estimates are in standard deviation (S.D.) units, with standard errors in brackets. WP is the effect of the average person deviating 1 S.D. unit from their own within-person mean score on the focal predictor (anxiety or depression). PM is the effect of the average individual scoring 1 S.D. unit higher on the person-mean variable of the focal predictor (anxiety or depression). For covariates, the estimates represent change in S.D. units for each unit change in the predictor. Estimates labeled a*b are interactions. All WP*covariate and PM*covariate interactions were included in the models, but non-significant interactions are not included in the table. Significance was based on standard errors.

* *p* < 0.05, ** *p* < 0.01, ****p* < 0.001

**Table 3S.** Associations between HADS anxiety and depression scores and performance on Logical memory (fixed effects)

|  | Basic model | Adjusted  for gender | Adjusted for  age 11 IQ | Adjusted for emotional stability | Adjusted for  no. of diseases | Fully adjusted model |
| --- | --- | --- | --- | --- | --- | --- |
| **Anxiety** |  |  |  |  |  |  |
| Age | 0.0049 (0.0059) | 0.0047 (0.0060) | 0.0026 (0.0062) | 0.0031 (0.0061) | 0.0040 (0.0061) | 0.0005 (0.0065) |
| WP | -0.0122 (0.1166) | -0.0758 (0.1426) | 0.0055 (0.1217) | -0.0145 (0.1211) | -0.0564 (0.1213) | -0.0402 (0.1598) |
| PM | -0.0931 (0.0476) | -0.1106 (0.0761) | -0.0706 (0.0501) | -0.0504 (0.0640) | -0.0872 (0.0485) | -0.0029 (0.1007) |
| WP*age | 0.0140 (0.0166) | 0.0155 (0.0167) | 0.0130 (0.0175) | 0.0129 (0.0170) | 0.0199 (0.0173) | 0.0141 (0.0187) |
| PM*age | 0.0125 (0.0070) | 0.0127 (0.0071) | 0.0140 (0.0077) | 0.0148 (0.0074)* | 0.0136 (0.0072) | 0.0169 (0.0080)* |
| WP*PM | -0.0426 (0.0743) | -0.0519 (0.0753) | -0.1076 (0.0821) | -0.0296 (0.0856) | -0.0397 (0.0769) | -0.1657 (0.1021) |
| Gender (female) |  | -0.0032 (0.0899) |  |  |  | -0.0072 (0.1048) |
| Age 11 IQ |  |  | 0.0169 (0.0031)*** |  |  | 0.0180 (0.0035)*** |
| Emotional stability |  |  |  | 0.0136 (0.0070) |  | 0.0129 (0.0076) |
| No. of diseases |  |  |  |  | -0.0552 (0.0287) | -0.0612 (0.0338) |
| **Depression** |  |  |  |  |  |  |
| Age | 0.0055 (0.0060) | 0.0053 (0.0060) | 0.0018 (0.0063) | 0.0029 (0.0062) | 0.0039 (0.0062) | -0.0013 (0.0066) |
| WP | **-0.2116 (0.0944)*** | **-0.2490 (0.1103)*** | -0.1768 (0.0947) | -0.1733 (0.0947) | -0.1737 (0.0970) | -0.2136 (0.1143) |
| PM | 0.0148 (0.0477) | 0.0121 (0.0730) | 0.0112 (0.0491) | 0.0737 (0.0580) | 0.0278 (0.0484) | 0.1042 (0.0929) |
| WP*age | 0.0222 (0.0115) | 0.0233 (0.0117)* | 0.0240 (0.0117)* | 0.0176 (0.0116) | 0.0177 (0.0123) | 0.0181 (0.0126) |
| PM*age | 0.0040 (0.0075) | 0.0044 (0.0075) | 0.0045 (0.0077) | 0.0057 (0.0077) | 0.0029 (0.0076) | 0.0053 (0.0081) |
| WP*PM | -0.0644 (0.0579) | -0.0651 (0.0579) | -0.1259 (0.0582)* | -0.0704 (0.0604) | -0.0627 (0.0595) | -0.0996 (0.0637) |
| WP*emotional stability |  |  |  | -0.0106 (0.0052)* |  |  |
| Gender (female) |  | -0.0260 (0.0885) |  |  |  | 0.0207 (0.1030) |
| Age 11 IQ |  |  | 0.0173 (0.0031)*** |  |  | 0.0182 (0.0036)*** |
| Emotional stability |  |  |  | 0.0150 (0.0064)* |  | 0.0113 (0.0069) |
| No. of diseases |  |  |  |  | -0.0615 (0.0290)* | -0.0577 (0.0341) |

HADS, Hospital Anxiety and Depression Scale.

Estimates are in standard deviation (S.D.) units, with standard errors in brackets. WP is the effect of the average person deviating 1 S.D. unit from their own within-person mean score on the focal predictor (anxiety or depression). PM is the effect of the average individual scoring 1 S.D. unit higher on the person-mean variable of the focal predictor (anxiety or depression). For covariates, the estimates represent change in S.D. units for each unit change in the predictor. Estimates labeled a*b are interactions. All WP*covariate and PM*covariate interactions were included in the models, but non-significant interactions are not included in the table. Significance was based on standard errors.

* *p* < 0.05, ** *p* < 0.01, ****p* < 0.001

**Table 4S.** Associations between HADS anxiety and depression scores and performance on Letter fluency (fixed effects)

|  | Basic model | Adjusted for  gender | Adjusted for  age 11 IQ | Adjusted for emotional stability | Adjusted for  no. of diseases | Fully adjusted model |
| --- | --- | --- | --- | --- | --- | --- |
| **Anxiety** |  |  |  |  |  |  |
| Age | -0.0168 (0.0047)*** | -0.0168 (0.0047)*** | -0.0227 (0.0051)*** | -0.0182 (0.0048)*** | -0.0182 (0.0048)*** | -0.0232 (0.0052)*** |
| WP | 0.1007 (0.0926) | 0.0537 (0.1118) | 0.0532 (0.1000) | 0.1141 (0.0945) | 0.1109 (0.0959) | -0.0026 (0.1283) |
| PM | **-0.0974 (0.0453)*** | **-0.1437 (0.0731)*** | -0.0658 (0.0462) | -0.0916 (0.0595) | **-**0.0888 (0.0465) | -0.1637 (0.0919) |
| WP*age | -0.0046 (0.0132) | -0.0037 (0.0133) | -0.0028 (0.0144) | -0.0055 (0.0133) | -0.0093 (0.0137) | -0.0050 (0.0151) |
| PM*age | 0.0073 (0.0056) | 0.0075 (0.0056) | 0.0003 (0.0064) | 0.0073 (0.0058) | 0.0064 (0.0057) | 0.0014 (0.0065) |
| WP*PM | -0.0664 (0.0575) | -0.0739 (0.0584) | -0.1022 (0.0663) | -0.0747 (0.0655) | -0.0498 (0.0591) | -0.1002 (0.0811) |
| Gender (female) |  | 0.1208 (0.0876) |  |  |  | 0.0827 (0.0964) |
| Age 11 IQ |  |  | 0.0213 (0.0029)*** |  |  | 0.0202 (0.0032)*** |
| Emotional stability |  |  |  | 0.0117 (0.0066) |  | 0.0059 (0.0069) |
| No. of diseases |  |  |  |  | -0.0534 (0.0281) | -0.0239 (0.0310) |
| **Depression** |  |  |  |  |  |  |
| Age | -0.0129 (0.0048)** | -0.0131 (0.0048)** | -0.0160 (0.0051)** | -0.0142 (0.0048)** | -0.0138 (0.0049)** | -0.0160 (0.0053)** |
| WP | **-0.2195 (0.0736)**** | **-0.2810 (0.0857)**** | **-0.2131 (0.0764)**** | **-0.1928 (0.0732)**** | **-0.1874 (0.0758)*** | **-0.2261 (0.0913)*** |
| PM | -0.0748 (0.0454) | **-0.1810 (0.0701)*** | -0.0825 (0.0451) | -0.0178 (0.0539) | -0.0811 (0.0462) | **-0.1766 (0.0837)*** |
| WP*age | 0.0118 (0.0091) | 0.0139 (0.0092) | 0.0095 (0.0094) | 0.0085 (0.0090) | 0.0098 (0.0097) | 0.0061 (0.0102) |
| PM*age | -0.0067 (0.0060) | -0.0052 (0.0060) | -0.0080 (0.0063) | -0.0059 (0.0060) | -0.0073 (0.0061) | -0.0070 (0.0066) |
| WP*PM | 0.0248 (0.0442) | 0.0226 (0.0442) | 0.0179 (0.0462) | 0.0010 (0.0459) | 0.0224 (0.0455) | 0.0068 (0.0504) |
| PM*no. of diseases |  |  |  |  | 0.0575 (0.0256)* | 0.0613 (0.0272)* |
| Gender (female) |  | 0.0623 (0.0856) |  |  |  | 0.0308 (0.0938) |
| Age 11 IQ |  |  | 0.0215 (0.0029)*** |  |  | 0.0205 (0.0032)*** |
| Emotional stability |  |  |  | 0.0143 (0.0060)* |  | 0.0065 (0.0062) |
| No. of diseases |  |  |  |  | -0.0616 (0.0281)* | -0.0318 (0.0310) |

HADS, Hospital Anxiety and Depression Scale.

Estimates are in standard deviation (S.D.) units, with standard errors in brackets. WP is the effect of the average person deviating 1 S.D. unit from their own within-person mean score on the focal predictor (anxiety or depression). PM is the effect of the average individual scoring 1 S.D. unit higher on the person-mean variable of the focal predictor (anxiety or depression). For covariates, the estimates represent change in S.D. units for each unit change in the predictor. Estimates labeled a*b are interactions. All WP*covariate and PM*covariate interactions were included in the models, but non-significant interactions are not included in the table. Significance was based on standard errors.

* *p* < 0.05, ** *p* < 0.01, ****p* < 0.001
