# Supplementary material for: Effects of between-person differences and within-person changes in symptoms of anxiety and depression on older age cognitive performance
Source: Psychol Med. 2017 Oct 17;48(8):1350–8. doi: 10.1017/S0033291717002896 (PMC6088541; doi:10.1017/S0033291717002896)
Supplement: Supplementary file 1 [file S0033291717002896sup.zip › S0033291717002896sup001.docx]

**Effects of between-person differences and within-person fluctuations in symptoms of anxiety and depression on older-age cognitive performance**

**Table S1.** Sample characteristics according to wave for participants who stayed in the study for the entire follow-up period (*n* = 50)

|  | Wave 1 | | Wave 2 | | Wave 3 | | Wave 4 | | Wave 5 | |
| --- | --- | --- | --- | --- | --- | --- | --- | --- | --- | --- |
|  | mean | S.D. | mean | S.D. | mean | S.D. | mean | S.D. | mean | S.D. |
| Age | 79.15 | 0.58 | 83.51 | 0.56 | 86.66 | 0.39 | 90.15 | 0.09 | 92.16 | 0.36 |
| Gender, % female | 56.00 | |  |  |  |  |  |  |  |  |
| Age 11 IQ, *n* = 42 | 101.82 | 12.73 |  |  |  |  |  |  |  |  |
| Emotional stability, *n* = 49 | 24.96 | 8.45 |  |  |  |  |  |  |  |  |
| No. of diseases, *n* = 46 | 1.13 | 1.39 |  |  |  |  |  |  |  |  |
| HADSa (anxiety) | 4.86 | 2.86 |  |  | 5.10 | 3.49 | 4.46 | 3.14 | 5.06 | 3.09 |
| HADSd (depression) | 3.26 | 2.26 |  |  | 3.38 | 2.12 | 3.72 | 2.20 | 4.72 | 2.45 |
| HADSa: deviation from within-person mean | -0.00 | 0.47 |  |  | 0.07 | 0.56 | -0.12 | 0.44 | 0.06 | 0.44 |
| HADSd: deviation from within-person mean | -0.22 | 0.57 |  |  | -0.17 | 0.47 | -0.02 | 0.55 | 0.41 | 0.63 |
| Raven’s^a^ | 34.70 | 8.35 | 33.78 | 6.90 | 31.36 | 7.46 | 27.49 | 8.22 | 26.00 | 8.18 |
| Logical memory | 35.18 | 13.81 | 37.86 | 15.03 | 38.38 | 14.10 | 37.58 | 15.78 | 37.24 | 18.68 |
| Letter fluency | 43.70 | 12.57 | 44.36 | 12.75 | 43.74 | 12.49 | 42.54 | 14.12 | 40.82 | 12.98 |

S.D., standard deviation; HADS, Hospital Anxiety and Depression Scale.

^a^ Number of participants with data on Raven’s were 50 at Waves 1, 2, and 3, 49 at Wave 4, and 46 at Wave 5
